# Gains in Life Expectancy Associated with Higher Education in Men

**DOI:** 10.1371/journal.pone.0141200

**Published:** 2015-10-23

**Authors:** Govert E. Bijwaard, Frans van Poppel, Peter Ekamper, L. H. Lumey

**Affiliations:** 1 Netherlands Interdisciplinary Demographic Institute (NIDI-KNAW)/University of Groningen, the Hague, the Netherlands; 2 Department of Epidemiology, Mailman School of Public Health, Columbia University, New York, United States of America; 3 Molecular Epidemiology, Leiden University Medical Center, Leiden, the Netherlands; Institute of Psychiatry, UNITED KINGDOM

## Abstract

**Background:**

Many studies show large differences in life expectancy across the range of education, intelligence, and socio-economic status. As educational attainment, intelligence, and socio-economic status are highly interrelated, appropriate methods are required to disentangle their separate effects. The aim of this paper is to present a novel method to estimate gains in life expectancy specifically associated with increased education. Our analysis is based on a structural model in which education level, IQ at age 18 and mortality all depend on (latent) intelligence. The model allows for (selective) educational choices based on observed factors and on an unobserved factor capturing intelligence. Our estimates are based on information from health examinations of military conscripts born in 1944–1947 in The Netherlands and their vital status through age 66 (n = 39,798).

**Results:**

Our empirical results show that men with higher education have lower mortality. Using structural models to account for education choice, the estimated gain in life expectancy for men moving up one educational level ranges from 0.3 to 2 years. The estimated gain in months alive over the observational period ranges from -1.2 to 5.7 months. The selection effect is positive and amounts to a gain of one to two months. Decomposition of the selection effect shows that the gain from selection on (latent) intelligence is larger than the gain from selection on observed factors and amounts to 1.0 to 1.7 additional months alive.

**Conclusion:**

Our findings confirm the strong selection into education based on socio-economic status and intelligence. They also show significant higher life expectancy among individuals with higher education after the selectivity of education choice has been taken into account. Based on these estimates, it is plausible therefore that increases in education could lead to increases in life expectancy.

## Introduction

Early life family characteristics including education and socio-economic status are important predictors of adult health and mortality [1–3]. The mortality differences by education hold across many populations and are persistent over time [4]. This is not only true for developing countries, but also for highly developed countries with advanced health care systems and extensive social security [5]. In eight Western European countries, lower education was associated with higher mortality from all causes and from cardiovascular diseases, neoplasms, and external causes [6]. Even in an egalitarian country such as the Netherlands, the difference in life expectancy between individuals with no formal education beyond primary school and those with a university education is more than five years [7]. The background of these inequalities is not fully understood [8, 9].

The association between mortality and education may partly be explained by confounding factors such as intelligence, and socio-economic status that affect both education choices and mortality [10]. Lower intelligence as measured by standardized IQ tests is related to increased mortality [11–13]. Because educational attainment and intelligence are strongly correlated, it is difficult to separate their effects on mortality [14].

Studies based on natural experiments in education including changes in compulsory schooling laws may to some extent overcome the difficulty of separating true education effects from these confounding factors. Recent analyses of such natural experiments suggest that the direct effects of education on mortality may be limited or even absent [15–18]. It is possible therefore that the strong association between education and mortality is partly due to the incomplete control of confounding factors.

The effects of intelligence on mortality could be operating in several ways. Indirect effects can be expected if higher intelligence drives better education and improvements in Socio-economic Status (SES) in later life [19]. Direct effects are likely if more intelligent individuals do better in managing their diseases and in seeking appropriate treatment where necessary [11]. As an example of the latter, higher educated men are more able to comply with and maintain complex health regimens that are prescribed to deal with HIV [20] and sooner adapt to evolving medical technologies [21, 22]. Education and intelligence may also operate in tandem and be mutually reinforcing. Failure to control for intelligence in the education health relation biases the estimated effect [23] and a better understanding of this relation is needed to establish potential direct benefits of improvements in education on mortality.

The aim of this paper is to estimate gains in life expectancy associated with increased education. Our approach to account for the interdependence of intelligence, education, and socio-economic status, and their joint influence on mortality, is based on structural models developed by Conti and Heckman [24, 25] and expanded to allow for duration outcomes [26] and to allow for ordered rather than binary education strata.

## Materials and Methods

### Study population

Using anonymized extracts from examinations for military service in the Netherlands between 1962–1965, we followed 45,037 men selected from the national birth cohorts 1944–1947 [27]. These examinations are based on yearly listings of all Dutch male citizens aged around 18 years in the national population registers. The study was reviewed by the Institutional Review Board of the Columbia University Medical Center in New York, NY. The Board determined that studies on this study population do not meet the DHSS definition of ‘human subjects research’ and are exempt from IRB approval. In the Netherlands, the study does not need approval by Ethical Review Boards or by the National Data Protection Authority (College Bescherming Persoonsgegevens) as all study procedures are in compliance with Dutch privacy legislation and do not need the consent of the data subjects concerned or of their relatives. The study is based on population wide administrative records and not on patient records.

### Examination records

The examination records contain detailed information on demographic and socio-economic status, including education, father’s occupation, birth order, religion, the place of birth, and a standardized psychometric test battery with several measures of intelligence.

The principal intelligence measure included is the Raven Progressive Matrices, a nonverbal untimed test that requires inductive reasoning about perceptual patterns [28]. Because the test does not depend on reading, writing, or language skills and is easily administered and interpreted it is widely used to test military conscripts across the world. We used separate tests for Arithmetic and Language performance. Scores for all tests were grouped in six levels from 1 (highest) to 6 (lowest). The test scores are highly correlated with Pearson’s *r* values in the range of .63 to .76.

The education system in the Netherlands is characterized by the nominal number of years of schooling and by two parallel streams in the educational system- general academic and vocational. Streaming choices are made at the end of primary school. Students in the vocational stream cannot directly enter university. Students with more than twelve years of education will nearly always be in the academic stream [29, 30].

Conscripts’ education was classified in four levels [31]: primary school (primary, six years of schooling); lower vocational education (eight years of schooling); lower secondary education (ten years of schooling); and intermediate or higher vocational or academic education (higher, twelve or more years of schooling). Because conscripts were age 18 at examination the highest education group includes men who had just started university.

Father’s occupation was classified into five categories: professional and managerial workers; clerical, self-employed and skilled workers; farmers; semi-skilled workers including operators, process workers and shop assistants; and laborers and miners. Fathers with unknown occupations were classified separately.

Birth order was recorded as reported by the examinee.

Place of birth was categorized in four urbanization levels distinguishing rural communities (rural communities with 20% or more farming population), urbanized rural communities (rural communities with less than 20% farming population), towns (townships and cities with less than 100,000 inhabitants), and cities with populations of 100,000 or more.

Self-reported religion was classified as Roman Catholic, Protestant (Dutch Reformed or Calvinist), other or none.

### Follow-up

As described elsewhere [27] we traced all examinees through population register records and national death records to ascertain vital status. Follow-up was until January 1, 2011, by which time the oldest men born in 1944 had reached the age of 66 years. We identified 45,037 men for tracing at age 18, and ascertained vital status for 41,096 (91.2%) as per January 1, 2011. Among this group, 35,157 (85.5%) were alive and 5,939 (14.5%) had died. For 1,316 (2.9%), only a partial follow-up was possible due to emigrations or other reasons, and for 2,625 (5.8%) no follow-up was possible because of missing data. For this study, we excluded partly institutionalized conscripts who had attended special schools for the illiterate, handicapped, deaf-mute, or mentally retarded, and conscripts who had not completed schooling through 12 years. After exclusion of these 2,614 conscripts, 39,798 men remain for analysis.

Our study is based on military examination records for men born in the Netherlands in the years 1944–1947 which were originally sampled to the study of the relation between prenatal famine exposure and mortality [27]. For that reason, men born in the Western Netherlands in 1944–1945 were over-represented. In this population, there was no relation however between famine conditions around birth and either mental performance [32] or education at age 18 [27]. The study population appears suitable therefore to obtain unbiased estimates of the relation between cognitive ability, education, and mortality.

### Data description

Selected demographic and socio-economic characteristics at the time of medical examinations are given in Table 1. Education level is strongly related to father’s occupation; men with the highest education tend to have fathers in the professional or managerial occupations. First-born conscripts also tend to have higher levels of education. In Table 2, intelligence test scores obtained by three instruments are presented by level of education. Again, men with the highest education tend to do best on all psychometric tests.

**Table 1:** Population characteristics at age 18 years by level of education.

	**Education level**			
	**Primary**	**Lower vocational**	**Lower secondary**	**Higher**
	n = 5,712	n = 14,572	n = 13,124	n = 6,390
	14%	36%	33%	16%
Birth order				
1	11%	33%	37%	19%
2	13%	37%	34%	16%
3	15%	39%	31%	14%
4	19%	40%	27%	13%
5+	24%	41%	26%	10%
Religion				
Roman Catholic	17%	36%	31%	16%
Protestant	12%	37%	34%	16%
Other	7%	36%	37%	20%
No Religion	15%	36%	33%	16%
Place of birth				
Rural	13%	42%	29%	17%
Urbanized Rural	13%	38%	33%	16%
Town	15%	34%	31%	20%
City	15%	35%	35%	16%
Father’s occupation				
Professional	7%	22%	34%	37%
Clerical	8%	31%	41%	20%
Farmer	3%	59%	21%	8%
Semi-skilled	12%	45%	29%	6%
Laborer	26%	44%	25%	4%
Unknown	19%	39%	30%	11%

Each row sums to 100%.

**Table 2:** Intelligence scores at age 18 years by level of education.

	**Education level**			
	**Primary**	**Lower vocational**	**Lower secondary**	**Higher**
	n = 5,712	n = 14,572	n = 13,124	n = 6,390
Raven score				
1 highest	2%	25%	40%	33%
2	7%	37%	38%	17%
3	17%	46%	31%	7%
4	28%	45%	22%	4%
5	47%	38%	14%	1%
6 lowest	54%	29%	15%	1%
Arithmetic test score				
1 highest	0%	16%	34%	50%
2	1%	32%	46%	21%
3	7%	45%	43%	5%
4	23%	53%	23%	1%
5	48%	44%	8%	0%
6 lowest	71%	26%	2%	0%
Language test score				
1 highest	0%	8%	43%	48%
2	2%	25%	52%	21%
3	11%	54%	30%	4%
4	26%	62%	11%	1%
5	50%	45%	4%	0%
6 lowest	74%	23%	2%	0%
Test scores not				
available	18%	28%	33%	21%

Each row sums to 100%.

### Statistical analysis

We seek to evaluate the impact of education on life expectancy accounting for intelligence and other individual factors that influence both the education choice and mortality. A common characteristic of survival data, including time to death, is that not all individuals experience the event of interest during the observation period. Such right censoring makes inference based on means unreliable. Another characteristic is dynamic selection: those still alive at age 18 may not be a random selection of the original population of births. We therefore use the (mortality) hazard, or the force of mortality, as this effectively deals with these data characteristics. A common approach would be to ignore the impact of intelligence and estimate a proportional hazard model for the mortality rate including the education level as one of the explanatory variables that proportionally changes the mortality rate. However, it is very likely that not only the scale of the mortality hazard but also the shape is affected by education. It is also plausible that the effect of other individual factors differs by education level. However, it is very likely that not only the scale of the mortality hazard but also the shape is affected by education. It is also plausible that the effect of other individual factors differs by education level. For example, having a father with a professional occupation is likely to have a larger impact for men with only primary education than for men with higher education. We therefore start our analyses with *separate proportional Gompertz* models, that is separate by education level. We choose a Gompertz mortality rate [33–35], which assumes an exponential increase in the mortality by age, because this is known to provide accurate mortality hazards for middle aged individuals [36].

**Fig 1:**
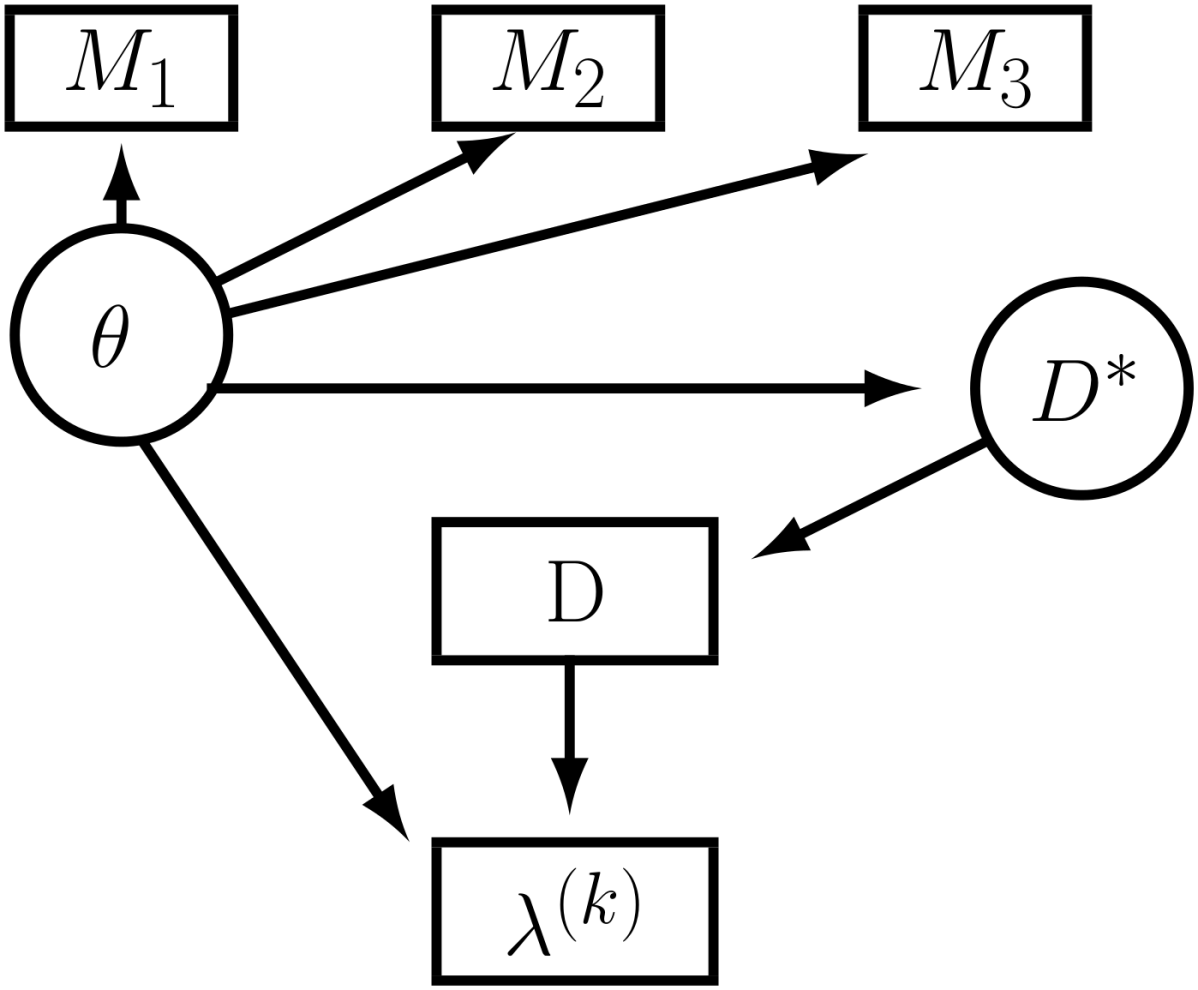
Schematic depiction of the structural equation model. Latent intelligence *θ* influences the utility, D*, of an individual choosing a particular education level, *D*. It also influences directly the (potential) mortality hazard *λ* ^(*k*)^, for each education level *D* = *k*. The value of three measured IQ-tests, *M*_1_, *M*_2_ and *M*_3_ all depend on the latent intelligence.

The difference in the implied life expectancy for these separate Gompertz models will only provide the educational gains in life expectancy accounting for observed individual differences by education. Individual intelligence seems an important factor influencing both the education choice and mortality and ignoring it would bias the results. However, intelligence is also heavily affected by parental background and by the education attained up till age 18 [37]. Another issue is that standard IQ-test are only a proxy of intelligence, or cognitive ability. This precludes the simple addition of intelligence as measured by IQ-test(s) into the Gompertz hazards. We therefore formulated a structural model to account for the interdependence of intelligence and education and their joint influence on mortality [26], extending the structural equation model of Conti and Heckman [24, 25] to a Gompertz proportional hazard model. The model allows for potential interdependencies between educational choice, intelligence and mortality. We assume that individuals base their schooling decision on expected utility maximizing and that the individual utility of each education level attained depends on the income earned and perceived health. This implies that the decision to continue schooling and, to attain a higher education level depend on expected income and (potentially) also on perceived health gains. The model consists of three parts: (i) the education choice, (ii) potential mortality hazards and (iii) a measurement system for latent intelligence. Fig 1 shows the structure of the model.

The education choice is based on maximizing the expected future utility. Let $D_{i}^{*}$ denote the (latent) utility of an individual choosing a particular education level. We assume that the educational choice is endogenous and that selection into schooling is fully accounted for by the observed individual characteristics and their latent cognitive ability. Define the indicator of education, *D*, taking the value *k* if the individual has attained education level *k* (1, …, 4): *D* = *k* if *ζ*_*k*−1_ < D* ≤ *ζ*_*k*_ with D* = *γ*′ *X* + *α*_*D*_ *θ* + *ν*_*D*_, the underlying latent utility of choosing a particular education level, which is continuous and depends linearly on the (vector of) observed characteristics *X* and latent intelligence *θ* and where *ζ*_0_ = −∞ and *ζ*_4_ = ∞. We assume that *ν*_*D*_ is normally distributed and assume an ordered probit model for the educational choice. Therefore the probability that an individual has attained education level *k* Pr(*D* = *k*) is given by

$$\Phi \left(\zeta_{k}-\gamma^{\prime }X-\alpha_{D}\theta \right)-\Phi \left(\zeta_{k-1}-\gamma^{\prime }X-\alpha_{D}\theta \right),$$ (1)

with Φ(⋅) as the standard normal cumulative density. Once the individual has decided his education level, future mortality is potentially causally related to this decision.

The second part of the structural model comprises the potential mortality hazards. These hazards are potential because each individual’s mortality is only observed for the actual education level and not for potential alternatives in education level. For each education level we choose a Gompertz mortality rate. Let *t* be the age of the individual, with the potential hazard for education level *k* $\lambda^{\left(k\right)}\left(t\right)=\exp\left(a_{k}t+\beta_{k0}+\beta_{k}^{\prime }X+\alpha_{k}\theta \right)$ depending on the (vector of) observed characteristics *X* and latent intelligence *θ*. The shape of the hazard is captured by *a*_*k*_ and the scale of the hazard by *β*_*k*0_. The effect of latent intelligence on the mortality hazard is captured by *α*_*k*_. The corresponding potential survival rates are

$$\begin{array}{ccc} S^{\left(k\right)}\left(t|X,\theta \right) & = & \exp(-\int_{0}^{t}\;\lambda^{\left(k\right)}\left(s|X,\theta \right)ds) \end{array}$$ (2)

$$\begin{array}{c} =\exp(-\frac{1}{a_{k}}e^{\beta_{k0}+\beta_{k}^{\prime }X+\alpha_{k}\theta }(e^{a_{k}}-1)) \end{array}$$ (3)

The Gompertz survival models for each of the four education levels include birth order, place of birth, religion, and father’s occupation as relevant control covariates related to survival and the impact of the latent intelligence. These models have a proportional hazard conditional on the latent intelligence. This is similar to including a (log-normal) frailty into the proportional hazard model [38].

The model is closed by three measurement equations linking the intelligence scores with the latent cognitive ability and the (vector of) observed individual characteristics. Because for each IQ test, *q* = 1, 2, 3, the continuous score is only observed in six ordered IQ classes, an ordered probit is assumed for each test with $M^{*}=\delta_{q}^{\prime }X+\alpha_{M_{q}}\theta +\nu_{M_{q}}$ where *ν*_*M*_*q*__ is normally distributed and *M*_*q*_ = *m* if *ϑ*_*q*_*m*−1__ < M* ≤ *ϑ*_*q*_*m*__. The probability to observe an individual in one of the IQ-classes is given by

$$\mathrm{Pr}\left(M_{q}=m\right)=\Phi \left(\vartheta_{q_{m}}-\delta_{q}^{\prime }X-\alpha_{M_{q}}\theta \right)-\Phi \left(\vartheta_{q_{m-1}}-\delta_{q}^{\prime }X-\alpha_{M_{q}}\theta \right).$$ (4)

An important feature of mortality outcomes is that some individuals are still alive at the end of the observation period and are right censored. Another feature of our data is that all men were about 18 years old at the start of the observation period at the time of military examination. We therefore condition on survival through the age at military examination. With the distribution assumptions on the educational choice, the latent mortality hazards and the measurement system the likelihood function is defined. We use a maximum likelihood estimation method to estimate all the parameters of the model. Details of the estimation procedure are presented in the Supporting Information. For the structural model, we jointly estimate the parameters of the education choice, the Gompertz-hazards and the measurement equations. We also estimate separate Gompertz proportional hazard models, in which we ignore the dependence on latent cognitive ability. From the estimated parameters the implied survival function can be calculated for each individual, given his observed characteristics *X* based on Eq (3). The results from the separate Gompertz proportional hazard models are used to decompose the educational gain into a selection on observed factors and on the latent intelligence.

At the individual level, the main questions relate to the difference in life expectancy after age 18 and to the difference in expected years lived between ages 18 and 66 (the minimum and maximum observed age) comparing two adjacent educational levels. For the calculation of the life expectancy we assume that the estimated Gompertz hazards hold throughout adult life starting at age 18 and extending beyond the highest observed age of 66. For the calculation of the expected years lived we only focus on the interval of observed ages. Both measures can be obtained by integrating the difference in survival, *S* ^(*k*+1)^(*t*) − *S* ^(*k*)^(*t*), over the relevant age interval [38], where *S* ^(*k*+1)^(*t*) denotes the survival time up to age *t* for an individual with education (*D* = *k* + 1), and *S* ^(*k*)^(*t*) is the survival time up to age *t* for those with education (*D* = *k*), for *k* = 1, …, 3. As we only observe the mortality of an individual for his chosen education level and not for the counterfactual higher or lower education level, we can only calculate the expected life-expectancy and expected years lived averaged over all (relevant) individuals. It is important to note that the relevant distribution of the observed characteristics and the latent cognitive ability differ by education level. Except for the lowest and the highest education level we can for each education level calculate estimates of three average life-expectancy/expected years lived: (*i*) using the distribution of the characteristics for the adjacent lower education level, (*ii*) using the distribution of the characteristics for the particular education level, and (*iii*) using the distribution of the characteristics for the adjacent higher education level.

To obtain the average gain in life expectancy we first calculate the average survival gain. The gain in survival $G_{ATU}^{k}\left(t\right)$ at age t when the educational level improves from level *k* to *k* + 1 with *k* = 1, 2, 3; referring to primary, lower vocational, lower secondary, and higher education is:

$$G_{ATU}^{k}\left(t\right)=\int \int E\;[S^{\left(k+1\right)}\left(t|X=x,\theta =c\right)-S^{\left(k\right)}\left(t|X=x,\theta =c\right)]\;dF_{X,\theta |D=k}\left(x,c\right)$$ (5)

where *X* are the included covariates and *θ* is the value of the latent intelligence. We integrate over the joint distribution of the covariates and the latent intelligence given education level *k* (the lowest of the two adjacent education levels) *F*_*X*,*θ*∣*D* = *k*_(*x*, *c*) to obtain the *average treatment effect on the untreated* (ATU). This treatment effect provides the average educational gain for the lower educated if they had obtained a higher education level. When interested in the effect of increasing the education level the average treatment on the untreated is the most relevant measure from a policy perspective, as it provides the educational gains for the population whose education level can be increased. Other treatment effects, such as the average treatment effect and the average treatment effect on the treated, take a less relevant reference population (respectively, the whole population and the population who has already attained a higher education).

The integrals described above cannot be solved analytically as the dimension of the covariates *X* is too large. The comparison of the survival functions also involves the counterfactual of surviving at another education level. Therefore simulations are needed to approximate the survival differences. This is explained in more detail in the Supporting Information methodological S1 Appendix.

Our interest is not limited to estimating gains in life expectancy associated with moving up one education level. We also want to decompose the observed difference in life expectancy into treatment effects and selection effects. The latter comprise effects from differences in observed characteristics and from differences in the unobserved intelligence. A limitation is that all observations are limited to the ages 18–66 years. The implied differences in expected years lived between ages 18 and 66 are therefore only shown by education level.

The standard (non-parametric) estimator of the survival function is the product-limit estimator proposed by Kaplan and Meier [39], $\hat{S}=\prod_{t_{i}\leq t}\left[1-\frac{d_{i}}{Y_{i}}\right]$ with *d*_*i*_ is the number of deaths at age *t*_*i*_ and *Y*_*i*_ is the number of individuals who are at risk of dying at age *t*_*i*_. The surface under the Kaplan-Meier provides the (non-parametric) estimate of the expected years lived of the individuals in the sample over the observed age interval [38]. We calculate the Kaplan-Meier survival function and the implied expected years lived between ages 18 and 66 for each education level. The Kaplan-Meier estimate of the survival function does not account for any observed factors that influence the survival, except for the education level. The unconditional differences in expected years lived based on the Kaplan-Meier estimates therefore provide the total educational effect on the expected years lived, which is the sum of the educational gain and a selection effect. The selection effect of education is the gain in expected years lived by individuals selecting themselves into different education levels. This gain is caused by the fact that individuals with different education levels also differ in other aspects that influence their survival. We can decompose the unconditional differences in expected years lived based on the Kaplan-Meier curves into the educational gain from Eq (5) and a residual, which is a selection effect on the basis of cognitive ability and the other observable factors. Mathematically,

$$G_{KM}^{k}\left(t\right)=G_{ATU}^{k}\left(t\right)+\varepsilon^{k}(X,\theta )$$ (6)

where $G_{KM}^{k}\left(t\right)=E[\int (S^{\left(1\right)}\left(t\right)-S^{\left(0\right)}\left(t\right))\;dt]$ represents the unconditional differences in expected years lived from 18 to 66 from the Kaplan-Meier survival curve, $G_{ATU}^{k}\left(t\right)$ is the treatment effect in Eq (5) from the structural model and $\varepsilon^{k}(X,\theta )$ represents the selection effect on the basis of observable characteristics *X* and cognitive ability *θ*, all when the educational level improves from level *k* to *k* + 1. Note that this selection effect is the combination of actual selection bias and selection based on perceived gains of higher education. The *selection effect*, $\varepsilon^{k}(X,\theta )$, can be further decomposed into a selection on observables, *selection effect observed*, and a selection on latent intelligence, *selection effect intelligence*

$$\varepsilon^{k}(X,\theta )=G_{KM}^{k}\left(t\right)-G_{ATU}^{k}\left(t\right)=[G_{KM}^{k}\left(t\right)-G_{sep}^{k}\left(t\right)]+[G_{sep}^{k}\left(t\right)-G_{ATU}^{k}\left(t\right)]$$ (7)

where

$$G_{sep}^{k}\left(t\right)=\int E\;[S^{\left(k+1\right)}\left(t|X=x\right)-S^{\left(k\right)}\left(t|X=x\right)]\;dF_{X|D=k}\left(x\right),$$ (8)

is the gain in expected years lived based on the estimated separate Gompertz proportional hazard models, i.e. the models that ignore the influence of intelligence on the mortality. We integrate over the joint distribution of the covariates given education level *k* *F*_*X*∣*D* = *k*_(*x*).

All model estimations were carried out using STATA statistical software version 12. All the simulations to calculate the educational gains were obtained using R version 2.15.

## Results

**Fig 2:**
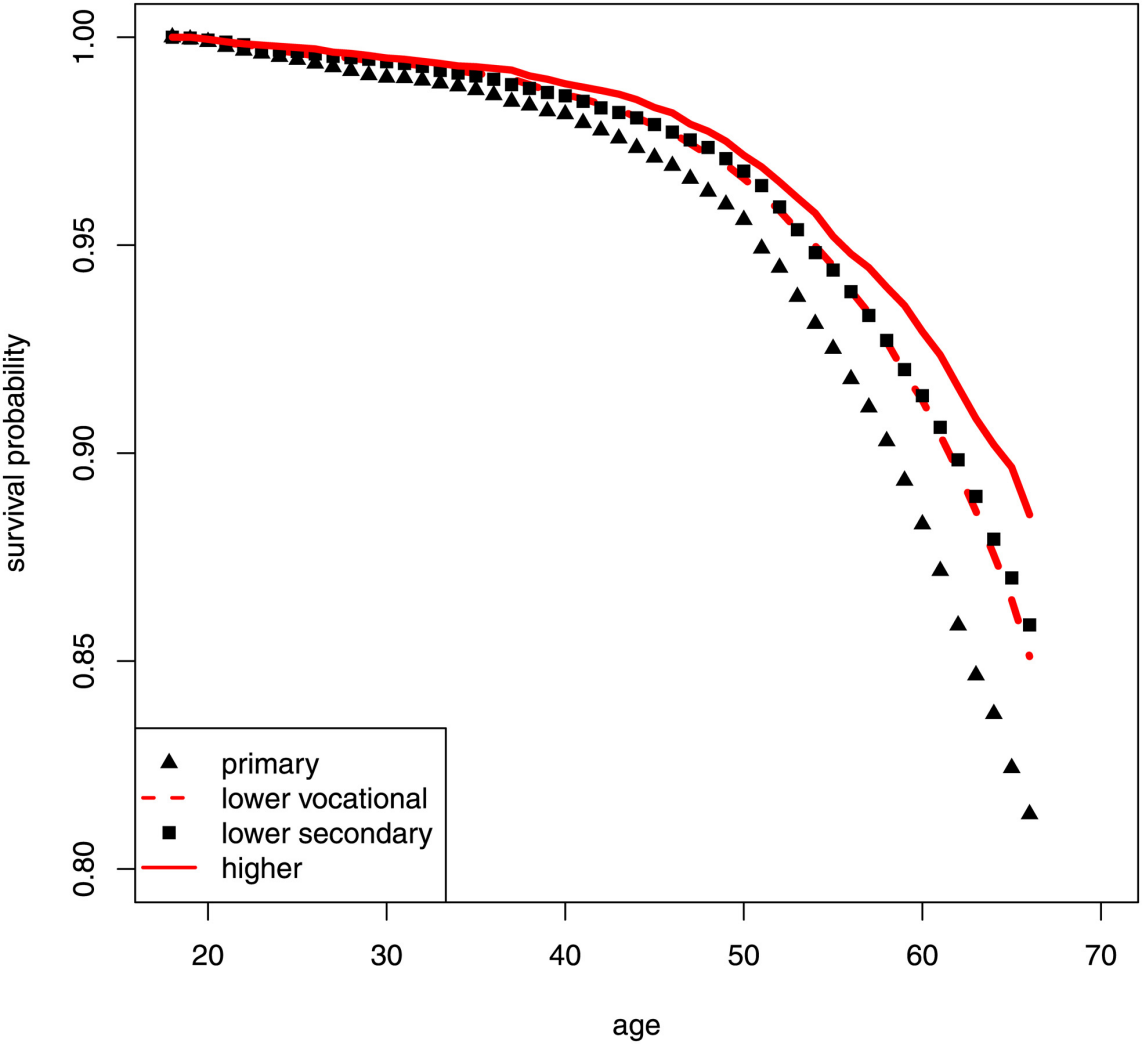
Kaplan-Meier estimates of survival by education level.

Survival increases with the education level and the differences increase with age. (*χ* ^2^ = 128.79 by log-rank test with 3 degrees of freedom). This can be seen from Kaplan-Meier survival curves for the four education categories primary, lower vocational, lower secondary, and higher education as shown in Fig 2. In subgroup analyses, survival differences comparing adjacent education levels are also statistically significant, except for survival in the lower vocational and lower secondary education groups that shows no difference (*χ* ^2^ = 1.91; d.f. = 1).

Men with lower vocational, lower secondary, and higher education have lower mortality compared to men with only primary education, with hazard ratios of 0.76 (95% CI 0.71 to 0.82), 0.73 (95% CI: 0.67 to 0.79), and 0.58 (95% CI: 0.52 to 0.64) respectively by Cox regression estimates. In subgroup analyses, men with lower vocational schooling show lower mortality compared to men with only primary education (HR 0.76; 95% CI: 0.71 to 0.82). Men with higher education show lower mortality compared to men with lower secondary education (HR 0.79; 95% CI: 0.73 to 0.87). There are no significant mortality differences between other adjacent education categories.

Ignoring for the moment the interdependence of the education choice, mortality and IQ-test scores, we first estimate Gompertz proportional hazard models separately for each of the four education levels. Table 3 presents the estimated hazard ratios. The results for the ordered probit models for the education choice and the IQ-tests are shown in S1 Table in the Supporting Information. As separate models are run for each education level, the impact of observed individual characteristics for each education level may differ but also the baseline hazard as reflected in the shape and the scale of the Gompertz hazard. It can indeed be seen that the scale and shape of the Gompertz mortality hazard differ substantially among the four educational groups (log-scale -9.572 to -10.149 and shape 0.083 to 0.090). Highly educated men born in urban areas have a lower mortality HR 0.72 (95% CI: 0.55 to 0.95) and men with only primary education also have a lower mortality when their fathers had a professional or managerial occupation HR 0.75 (95% CI: 0.57 to 0.99).

**Table 3:** Estimated mortality hazard ratios of separate proportional Gompertz models by education choice.

	**Education level**			
	**Primary**	**Lower vocational**	**Lower secondary**	**Higher**
Birth order	0.99 (0.96; 1.02)	0.98 (0.95; 1.00)	1.00 (0.97; 1.04)	0.99 (0.93; 1.05)
Religion (ref = without)				
Roman Catholic	1.03 (0.89; 1.20)	0.92 (0.82; 1.04)	0.97 (0.85; 1.10)	1.15 (0.94; 1.41)
Protestant	0.96 (0.81; 1.15)	0.98 (0.87; 1.10)	0.96 (0.85; 1.08)	1.00 (0.82; 1.23)
Other religion	0.73 (0.50; 1.06)	0.95 (0.80; 1.14)	0.83 (0.68; 1.00)	0.81 (0.59; 1.10)
Urbanization (ref = large city)				
Rural	0.90 (0.75; 1.08)	0.94 (0.84; 1.06)	0.91 (0.79; 1.04)	0.90 (0.73; 1.09)
Urbanized rural	1.02 (0.72; 1.45)	1.07 (0.85; 1.34)	0.99 (0.77; 1.29)	1.17 (0.80; 1.72)
Urban	0.86 (0.69; 1.07)	1.05 (0.90; 1.22)	1.05 (0.89; 1.24)	0.72* (0.55; 0.95)
Father’s occupation (Ref = white collar)				
Professional and managerial	0.75* (0.57; 0.99)	1.03 (0.88; 1.21)	0.99 (0.86; 1.13)	1.13 (0.95; 1.35)
Farm owners	0.93 (0.61; 1.43)	1.02 (0.82; 1.26)	1.11 (0.79; 1.55)	1.59 (0.95; 2.68)
Skilled laborers	0.97 (0.82; 1.16)	0.93 (0.83; 1.04)	1.09 (0.97; 1.23)	1.21 (0.92; 1.59)
Unskilled laborers	1.10 (0.91; 1.33)	0.94 (0.82; 1.08)	1.10 (0.93; 1.30)	1.30 (0.88; 1.92)
Unknown	0.99 (0.76; 1.30)	1.36* (1.14; 1.62)	1.02 (0.81; 1.27)	1.54* (1.08; 2.20)
scale (log)	-9.572 (-9.95; -9.20)	-10.089 (-10.36; -9.82)	-9.978 (-10.26; -9.70)	-10.149 (-10.59; -9.70)
shape	0.085 (0.079; 0.092)	0.090 (0.086; 0.095)	0.086 (0.081; 0.091)	0.083 (0.076; 0.091)

95% confidence interval within brackets. Significance: *p < 0.05

The structural models assumes that the three components of the model, the education choice, the IQ-measurements and the mortality hazards, are interdependent through the unobserved latent intelligence. Table 4 reports the hazard ratios for the mortality component, the odds ratios for the ordered probit education choice and the odds ratios of the ordered probit IQ-measurements. The first row of Table 4 shows that our latent factor of intelligence is strongly related to intelligence and education choice: the higher the intelligence, the higher the IQ score and the higher the attained education level. A one standard deviation higher intelligence increases the odds of higher education 3.09 times and the odds of higher IQ 4.02 (Raven), 6.88 (Arithmetic) and 6.62 (Language) times. Higher intelligence estimates show a lower mortality (HR: 0.76 to 0.90) for all education classes which is especially evident (and statistically significant) for the second (HR: 0.76; 95% CI: 0.70, 0.83) and third (HR: 0.85; 95% CI: 0.77, 0.94) education level. As shown in Table 1, men with fathers in professional or managerial positions are more likely to obtain higher education (OR: 1.54; 95% CI: 1.48, 1.60) and there is an education gradient by father’s level of occupation. Father’s occupation has a similar relation to the measures of intelligence (OR: Raven 1.16; Arithmetic 1.58; Language 1.58). The impact of father’s occupation on the mortality hazard within education level is variable.

**Table 4:** Estimated odds and hazard ratios for structural model.

	**Educational choice**	**Intelligence measurement**			**Mortality hazard**			
		Raven	Arith.	Language	Primary	Lower vocational	Lower secondary	Higher
Cognitive ability	3.09* (3.02; 3.16)	4.02* (3.92; 4.13)	6.99* (5.83; 8.38)	6.62* (5.44; 8.05)	0.90 (0.80; 1.01)	0.76* (0.70; 0.83)	0.85* (0.77; 0.94)	0.86 (0.71; 1.04)
Birth order	0.89* (0.88; 0.90)	0.93* (0.92; 0.94)	0.81* (0.79; 0.83)	0.78* (0.76; 0.80)	0.99 (0.96; 1.03)	0.98 (0.96; 1.01)	1.01 (0.97; 1.04)	0.99 (0.93; 1.05)
Religion (ref = without)								
Roman Catholic	0.99 (0.96; 1.03)	1.03 (0.99; 1.07)	1.24* (1.14; 1.34)	1.05 (0.97; 1.15)	1.04 (0.89; 1.21)	0.92 (0.82; 1.04)	0.97 (0.86; 1.10)	1.14 (0.93; 1.40)
Protestant	1.06* (1.02; 1.10)	0.99 (0.96; 1.03)	1.35* (1.24; 1.47)	1.25* (1.15; 1.36)	0.96 (0.81; 1.14)	0.98 (0.87; 1.10)	0.96 (0.85; 1.08)	1.00 (0.81; 1.22)
Other religion	1.31* (1.24; 1.39)	1.13* (1.07; 1.20)	2.15* (1.88; 2.45)	2.34* (2.04; 2.68)	0.73 (0.50; 1.05)	0.94 (0.79; 1.12)	0.81* (0.67; 0.98)	0.79 (0.58; 1.08)
Urbanization (ref = large city)								
Rural	1.01 (0.97; 1.05)	0.86* (0.83; 0.90)	0.98 (0.90; 1.07)	0.91 (0.84; 0.99)	0.90 (0.75; 1.07)	0.94 (0.84; 1.06)	0.91 (0.79; 1.04)	0.89 (0.73; 1.09)
Urbanized rural	0.96 (0.90; 1.04)	0.85* (0.79; 0.92)	0.92 (0.78; 1.10)	0.87 (0.73; 1.04)	1.01 (0.72; 1.43)	1.07 (0.85; 1.34)	1.01 (0.78; 1.30)	1.17 (0.80; 1.72)
Urban	1.05* (1.00; 1.10)	1.05* (1.00; 1.11)	1.36* (1.21; 1.52)	1.15* (1.03; 1.29)	0.85 (0.68; 1.06)	1.05 (0.90; 1.23)	1.05 (0.89; 1.24)	0.72* (0.55; 0.94)
Father’s occupation (Ref = white collar)								
Professional and managerial	1.54* (1.48; 1.60)	1.16* (1.11; 1.21)	1.58* (1.43; 1.75)	1.58* (1.43; 1.75)	0.74* (0.56; 0.97)	1.00 (0.85; 1.17)	0.97 (0.84; 1.12)	1.11 (0.93; 1.32)
Farm owners	0.53* (0.49; 0.57)	0.55* (0.50; 0.60)	0.30* (0.25; 0.37)	0.22* (0.18; 0.27)	0.94 (0.62; 1.44)	1.04 (0.84; 1.30)	1.17 (0.84; 1.65)	1.68 (0.99; 2.85)
Skilled laborers	0.45* (0.43; 0.46)	0.64* (0.61; 0.66)	0.24* (0.22; 0.26)	0.22* (0.20; 0.24)	1.00 (0.84; 1.19)	0.99 (0.88; 1.10)	1.14 (1.01; 1.29)	1.26 (0.96; 1.66)
Unskilled laborers	0.37* (0.36; 0.39)	0.54* (0.51; 0.57)	0.15* (0.13; 0.17)	0.15* (0.13; 0.17)	1.13 (0.94; 1.37)	1.01 (0.87; 1.16)	1.16 (0.98; 1.38)	1.39 (0.93; 2.07)
Unknown	0.55* (0.51; 0.58)	0.74* (0.69; 0.79)	0.30* (0.26; 0.35)	0.35* (0.30; 0.40)	1.01 (0.77; 1.32)	1.41 (1.18; 1.68)	1.06 (0.84; 1.33)	1.60* (1.12; 2.28)
scale (log)	-	-	-	-	-9.685 (-10.08; -9.29)	-10.211 (-10.48; -9.94)	-9.996 (-10.28; -9.72)	-10.079 (-10.53; -9.63)
shape	-	-	-	-	0.085 (0.079; 0.092)	0.090 (0.086; 0.095)	0.086 (0.081; 0.091)	0.083 (0.076; 0.091)

Raven: Raven progressive matrices test; Arith.: Arithmetic performance test; Language: language performance test. 95% confidence interval within brackets. Significance: *p < 0.05

Implicit in Table 4 is that the life expectancy in each education group not only depends on the education level but also varies by observed individual characteristics such as: birth order, religion, urbanization level and father’s occupation. As the distribution of these variables differs by education level, see Table 1, the averaging of the estimated Gompertz survival functions over the distribution of included variables will also vary by education level. We therefore calculate the average life expectancy implied by the estimated models averaging over the distribution of individual characteristics stratified by education level. These distributions, stratified by education level, are used to estimate the life expectancy (assuming the estimated Gompertz hazard holds beyond the highest observed age) and the expected years lived between age 18 and 66 years for the average person in that education level for (a) individuals who actually attained that level and for (b) individuals in the adjacent higher education level.

Table 5 presents the average life expectancy in years at age 18 by education level. The upper part of the table provides the life expectancies derived from the estimated simple stratified Gompertz models. These life expectancies at age 18 among men with the lowest to the highest education category range from 59.5 to 66.4 years (Table 5, upper part diagonal). There is a monotone, non-linear, relation between education level and life expectancy. Individuals with the highest education are expected to live, on average, almost six years longer than individuals with the lowest education. The educational gain on life expectancy (ATU) of an average individual moving one education level up can be obtained from comparing the life expectancy in the attained education level with the life expectancy, of the same individuals, one level higher, i.e. moving to the right in the table. The implied education gains on the life expectancy for men aged 18 are from primary to lower vocational education 2.2 years (61.7 minus 59.5 years) with a 95% CI from 0.6 to 3.8 years, from lower vocational to lower secondary education 1.4 years (95% CI: 0.1 to 2.7) and from lower secondary to higher education 2.9 years (95% CI: 1.0; 4.8).

**Table 5:** Life expectancy in years (95% CI) at age 18 by education level estimated from simple Gompertz and Structural Models.

	**Education level**			
	**Primary**	**Lower vocational**	**Lower secondary**	**Higher**
**Simple Gompertz**				
Factor distribution				
Primary	59.5 (59.5; 60.9)	61.7 (60.8; 62.6)		
Lower vocational		61.6 (60.8; 62.5)	63.0 (62.0; 64.0)	
Lower secondary			63.2 (62.2; 64.2)	66.1 (64.4; 67.7)
Higher				66.4 (64.7; 68.0)
**Structural model**				
Factor distribution				
Primary	60.6 (59.8; 61.3)	62.6 (62.1; 63.1)		
Lower vocational		62.6 (62.1; 63.1)	62.9 (62.4; 66.0)	
Lower secondary			63.2 (62.7; 63.6)	65.1 (64.2; 66.0)
Higher				65.6 (64.7; 66.5)

95% confidence interval within brackets.

The lower part of Table 5 provides the life expectancies implied by the structural model stratified by education level. Using the structural model the differences in life expectancy by education level are smaller than using simple stratified Gompertz models. The structural model accounts for the cognitive ability that both influences educational choice and mortality. This leads to a higher life expectancy estimate for the lowest education level and a lower estimate for the highest education level. Nevertheless, education level still plays a dominant (and significant) role in mortality; substantial differences in life expectancy are implied by changes in the coefficients of the mortality hazard. Based on our model, men with the lowest level of education might have lived 2 years longer (62.6 vs 60.6 years; 95% CI: 1.0 to 2.9) had they attained the next higher level of lower vocational education. The implied gain in life expectancy for men with lower vocational education had they attained the next higher level of lower secondary education is 0.3 years (95% CI: -0.4 to 1.0). Men with lower secondary education might have gained 1.9 years of life (95% CI: 0.9 to 2.9) had they attained higher education. Differences in observed individual characteristics explaining the education choice appear to be less important as can be seen from comparing the life expectancies within columns. As an example, there is little change in the life expectancy of individuals with lower vocational education moving to lower secondary education if the factor distribution of this group is applied.

In Table 6, we decompose the estimated gains in life expectancy between 18–66 years from the Kaplan-Meier curves into treatment and selection effects. The estimated treatment gains (column 1) are expressed as additional months lived by a shift to the next education level. We report gains in months because the gains in life expectancy (average months lived) are limited to the observation window 18 to 66 years. The estimated gains are 5.7 (95% CI: 4.2 to 7.2) additional months moving from the primary to the lower vocational education level, -1.2 months moving from lower vocational to lower secondary level, and 1.4 months (95% CI: 0.2 to 2.7) moving from lower secondary to higher education. For the intermediate education group with lower vocational education the treatment effect of education is negative. The selection effect, i.e. the positive impact on months lived for individuals selecting themselves into the next higher education level, is positive for all education levels. It amounts to one to two additional months of life (Table 6, column 2).

**Table 6:** Decomposition of the estimated gains in expected months lived from age 18 to 66 years into treatment and selection effects, on observables and selection on cognitive ability.

	**Treatment effect**	**Selection effect**		
		**Total**	**Observed**	**Intelligence**
From primary to lower vocational	5.7* (4.2; 7.2)	1.0 (-0.6; 2.5)	-0.1 (-0.6; 0.5)	1.0 (-0.5; 2.5)
From lower vocational to lower secondary	−1.2* (-2.1; -0.2)	1.4* (0.4; 2.4)	-0.3 (-0.7; 0.1)	1.7* (0.8; 2.7)
From lower secondary to higher	1.4* (0.2; 2.7)	2.1* (0.7; 3.4)	0.7* (0.3; 1.2)	1.3* (0.0; 2.6)

Change in expected months lived over the age range 18–66 years. Treatment effect: estimated gain from structural model. Selection effect: remaining difference from implied life expectancy based on Kaplan-Meier curves. Observed: selection effect due to difference in observed individual characteristics. Intelligence: selection effect due to difference in intelligence. 95% confidence interval within brackets. Significance: *p < 0.05

Next we decompose the selection effect of moving up one education level into two components: the first is attributable to selection on observed individual characteristics and the second to selection on intelligence. (Table 6, columns 3 and 4). The latter effect is predominant and amounts to 1.0 month additional living moving from primary to lower vocational education, 1.7 additional months moving from lower vocational to lower secondary education, and 1.3 additional months moving from lower vocational to higher education.

## Discussion

Our findings confirm a strong selection into education based on individual socio-economic status and intelligence. This is in agreement with previous findings from the United States where the correlation between IQ scores and years of education is about 0.55, and years of education are also positively correlated with the occupation/education of a child’s parents [28]. We therefore used a structural model to estimate the independent and combined effects of education and intelligence on life expectancy and show that improvements in education alone are likely to have a significant beneficial impact on life expectancy. These findings are more optimistic than previous reports suggesting that the direct effects of education on mortality may be limited or even absent [15–18].

Our contribution to the literature is twofold. First, we use an innovative structural model to estimate educational gains in life expectancy accounting for interdependence of intelligence and education and their joint influence on mortality. Second, we apply the model to a large nationally representative study population to obtain accurate and unbiased effect estimates.

The overall mortality from age 18 to the end of the observation period in our study was 12.7%, in close agreement with national estimates based on cohort life tables [27]. For 5.8% of men it was not possible to ascertain vital status at any point in time due to the lack of linkages across databases of information collected at age 18 with current data. These men could have died or could still be living in the Netherlands. As the traced and untraced men did not differ with respect to available demographic and examination characteristics at age 18 we nevertheless think that our study population provides unbiased effect estimates of the likely benefits of increased education on mortality.

The Raven test is widely used to test military conscripts across the world for its ease of administration and interpretation. At the age of 18, the examinees can be also assumed to have reached the peak of their problem solving skills on the tests of mental performance [40]. There has been a significant increase over time in the mean Raven scores of Dutch military recruits. In 1952, only 0.38% of Dutch recruits had IQs over 140 but this proportion had increased to 9% in 1982 [41]. The reasons for this score increase over time are unclear [40]. IQ score changes over time are not likely to impact on our study findings however because all IQ measurements were completed in a narrow time window of less than 3 years and the age at examination for the study population did not change. Although the study group was closely matched on both year of birth and year of examination of IQ scores we nevertheless evaluated the effects of additional adjustments for year of birth and year of examination to exclude any time-related trends and found no changes from our reported estimates.

Although the proportion of individuals lost to follow-up is relatively small, we carried out sensitivity analyses comparing the extreme scenarios under which all individuals lost to follow-up were either assumed to be dead or alive. This did not change the obtained treatment and selection effects. Assuming that missing individuals are in fact alive does increase the calculated life expectancies, but this had no impact on the reported survival differences associated with education levels.

A drawback of our data is that only information on men and not on women is available. Using survey data from a slightly older cohort Bijwaard et al. [26] found that educational gains for women appear to be higher than for men, in spite of the higher survival difference of women with lower vs higher education. However, these findings are based on much smaller numbers than the current study and only use data from one particular province, which is not necessarily representative for the whole country, and therefore need to be interpreted with caution.

The study findings apply to men born 1944–1947 who were examined in the early 1960s and our specific mortality findings may therefore not apply to contemporary populations in the Netherlands. There has been a major change in the education system in the Netherlands in 1968 and some of the specific education strata in this study no longer exist [30]. In addition, the percentage of people with more than six years of post-primary school education is currently much higher compared to the past [42]. These changes are not likely to affect our general conclusion that increased education can improve survival, but further long term studies will be needed to quantify these effects for contemporary school types.

In the shorter term, our models can be used to estimate the direct effects of education changes on health outcomes in contemporary populations. In many countries, including the Netherlands, annual individual level educational test scores are collected nationwide from kindergarten onwards. Of special importance from a policy perspective would be the analysis of health outcomes by ethnicity in view of the underrepresentation of many ethnic populations in the higher levels of national education systems. These analyses can further clarify the role of education on health and mortality in specific population groups.

In conclusion, our study aim was to assess the potential gains in life expectancy from increased education accounting for differences in intelligence and found significant positive gains. Our findings confirm the strong selection into education based on socio-economic background and on intelligence but nevertheless show significant beneficial gains from increased education.

For future research, we hope that similar analyses will be carried out in other countries with comparable data. Comparisons of study findings may then allow further specifications of the impact of education on survival inequalities over time. The continued analysis of this aging study population in the Netherlands will provide more refined estimates of education effects on survival, including cause-specific mortality, to the large group of men who live beyond the age of 66 years.

## Supporting Information

S1 Appendix (PDF)

S1 Table (PDF)
