# Supplementary material for: Gains in Life Expectancy Associated with Higher Education in Men
Source: PLoS One. 2015 Oct 23;10(10):e0141200. doi: 10.1371/journal.pone.0141200 (PMC4619701; doi:10.1371/journal.pone.0141200)
Supplement: S1 Appendix — (PDF) [file pone.0141200.s001.pdf]

Gains in life expectancy associated with higher education in men

Govert E. Bijwaard[1,*], Frans van Poppel[1], Peter Ekamper[1], L.H.Lumey[2,3]

1 Netherlands Interdisciplinary Demographic Institute (NIDI-KNAW)/University of Groningen, the Hague, the Netherlands
2 Department of Epidemiology, Mailman School of Public Health, Columbia University, New York, USA
3 Molecular Epidemiology, Leiden University Medical Center, Leiden, the Netherlands

*** Bijwaard@nidi.nl**

Supporting Information

S1 Appendix

Here we discuss some additional methodological issues involved in the structural model.

Combining the distribution assumptions on the educational choice, the latent mortality hazards and the measurement system allows us to write down the likelihood function. The likelihood contribution of individual i with education level k in our survival model is

$$L_i^{(k)} = \lambda^{(k)}(t)^{\Delta_i} S^{(k)}(t)/S^{(k)}(18), \qquad k = 1, \ldots, 4 \tag{S.1}$$

with $\Delta_i = 1$ if individual i has died before the end of the observation period, and zero otherwise. The (potential) survival functions are given in (1). With left-truncated data the distribution of latent cognitive ability among the survivors (up to the left-truncation time) changes. When only individuals are observed that have survived until age 18 the likelihood contribution is

$$\begin{aligned} L_i \quad = \quad & \int \prod_{k=1}^{4} \Bigg[\Big[\Phi(\zeta_k - \gamma X - \alpha_D \theta) - \Phi(\zeta_{k-1} - \gamma X - \alpha_D \theta) \Big] \\ & \qquad \times \lambda^{(k)}(t|X,\theta)^{\Lambda_i} S^{(k)}(t|X,\theta)/S^{(k)}(18|X,\theta) \Bigg]^{I_{ki}} \\ & \times \prod_{q=1}^{3} \prod_{m=1}^{6} \Big[\Phi(\vartheta_{q_m} - \delta_q X - \alpha_{M_q} \theta) - \Phi(\vartheta_{q_{m-1}} - \delta_q X - \alpha_{M_q} \theta) \Big] \\ & \qquad dH(\theta|T > 18) \end{aligned} \tag{S.2}$$

with $I_{ki} = 1$ when individual i has education level k and zero otherwise, and with the distribution of the latent abilities conditional on survival up to age 18

$$dH(\theta|T > 18) = \frac{\prod_{k=1}^{4} \Big[\Big[\Phi(\zeta_k - \gamma X - \alpha_D \theta) - \Phi(\zeta_{k-1} - \gamma X - \alpha_D \theta) \Big] S^{(k)}(18|X,\theta) \Big] h(\theta)}{\int \prod_{k=1}^{4} \Big[\Big[\Phi(\zeta_k - \gamma X - \alpha_D \theta) - \Phi(\zeta_{k-1} - \gamma X - \alpha_D \theta) \Big] S^{(k)}(18|X,\theta) \Big] h(\theta)\, d\theta} \tag{S.3}$$

with $h(\theta)$ is a normal distribution with variance $\sigma_\theta^2 = 1$.

Unfortunately, estimation based on the maximum of the likelihood function involves the calculation of an integral that does not have an analytical solution. This one dimensional integral can be approximated very well using a Gaussian quadrature, which is a numerical integration method based on Hermite polynomials [1]. Bijwaard et al. [2] provide a full description of the estimation of a similar model. The only difference is that we have a system of ordered probit measurements.

The integral in calculating the gain in survival $G^k_{ATU}(t)$ cannot be solved analytically, we therefore resort to a simulation procedure to obtain the survival gains and the life expectancies. For each education level we simulate the survival of 10,000 individuals. To each individual we assign observed characteristics based on the empirical distribution in the sample. The simulation procedure consists of five steps:

1. Draw a vector of parameter estimates assuming that the estimator is normally distributed around the point estimates with a variance-covariance matrix equal to the estimated one.
2. Compute the conditional hazard rates based on these parameter values and individual characteristics using the two Gompertz mortality hazard rates ($D = k$ and $D = k + 1$) conditional on the value of the latent cognitive ability.
3. Determine the unconditional survival function for every individual and for the whole age-range from 18 to 110 and by integrating out the latent cognitive ability through Gaussian quadrature methods.
4. Calculate the average (over the 10,000 individuals) survival at each age (with steps of a month).
5. Calculate the life expectancy from the surface under the survival function.

We repeat these steps 100 times to obtain 100 independent observations of the life expectancy for each education level. Bijwaard et al. [2] provide the details of this procedure.

Without additional restrictions on the distribution of the latent factors the model is not identified. The restrictions required for identification differ however from the restrictions on the latent factors in a standard structural equations model. In our case, an intrinsically non-linear duration outcome is modeled instead of a linear outcome. Identification of our model is closely related to the identification in a mixed proportional hazard (MPH) model, where the unobserved heterogeneity is assumed to have a log-normal distribution. An MPH model is identified when the unobserved heterogeneity term has a finite mean and is independent of the other observed factors [3]. We assume a normal distribution for the latent cognitive skills. For identification we set the variance of θ to one [2]. Thus the latent intelligence are assumed to follow a standard normal distribution.

References

1. Press WH, Flannert BP, Teukolsky SA, Vetterling WT. Numerical Recipes in C: The Art of Scientific Computing (second edition). Cambridge: Cambridge UP; 1993.
2. Bijwaard GE, van Kippersluis H, Veenman J. Education and Health: the Role of Cognitive Ability. Journal of Health Economics. 2015;forthcoming.
3. Elbers C, Ridder G. True and spurious duration dependence: The identifiability of proportional hazards models. Review of Economic Studies. 1982;49:403–410.
