# Supplementary material for: Gains in Life Expectancy Associated with Higher Education in Men
Source: PLoS One. 2015 Oct 23;10(10):e0141200. doi: 10.1371/journal.pone.0141200 (PMC4619701; doi:10.1371/journal.pone.0141200)
Supplement: S1 Table — (PDF) [file pone.0141200.s002.pdf]

Gains in life expectancy associated with higher education in men

Govert E. Bijwaard[1,*], Frans van Poppel[1], Peter Ekamper[1], L.H.Lumey[2,3]

1 Netherlands Interdisciplinary Demographic Institute (NIDI-KNAW)/University of Groningen, the Hague, the Netherlands
2 Department of Epidemiology, Mailman School of Public Health, Columbia University, New York, USA
3 Molecular Epidemiology, Leiden University Medical Center, Leiden, the Netherlands

*** Bijwaard@nidi.nl**

Supporting Information

S1 Table

Table S.1 presents the estimated odds ratios (OR) for the ordered probit estimation of the education choice and for the ordered probit estimation for each IQ test.

Table S.1. Estimated odds ratios in separate ordered probit models for education choice and for IQ-tests.

	**Education**	**IQ-test**		
		Raven	Arith	Language
Birth order	0.89*	0.95*	0.93*	0.92*
	(0.91; 0.92)	(0.95; 0.96)	(0.93; 0.84)	(0.92; 0.93)
Religion (ref = without)				
Roman Catholic	0.99	1.02	1.06	1.02
	(0.96; 1.02)	(0.99; 1.05)	(1.04; 1.09)	(0.99; 1.04)
Protestant	1.05*	1.00	1.10	1.07
	(1.02; 1.08)	(0.97; 1.02)	(1.07; 1.13)	(1.04; 1.10)
Other religion	1.23*	1.09	1.26	1.30
	(1.18; 1.29)	(1.05; 1.14)	(1.21; 1.32)	(1.25; 1.36)
Urbanization (ref=large city)				
Rural	1.01	0.90	0.99	0.97
	(0.98; 1.04)	(0.87; 0.92)	(0.96; 1.02)	(0.94; 0.99)
Urbanized rural	0.99	0.91	1.00	0.96
	(0.93; 1.04)	(0.86; 0.96)	(0.94; 1.05)	(0.91; 1.01)
Urban	1.04*	1.04*	1.10*	1.04
	(1.00; 1.08)	(1.00; 1.07)	(1.06; 1.14)	(1.01; 1.08)
Father's occupation (Ref=white collar)				
Professional and managerial	1.41*	1.11*	1.15*	1.15*
	(1.37; 1.46)	(1.07; 1.14)	(1.12; 1.19)	(1.12; 1.19)
Farm owners	0.61*	0.65*	0.69*	0.62*
	(0.58; 0.65)	(0.62; 0.69)	(0.65; 0.73)	(0.59; 0.66)
Skilled laborers	0.53*	0.73*	0.64*	0.61*
	(0.52; 0.55)	(0.71; 0.75)	(0.62; 0.65)	(0.60; 0.63)
Unskilled laborers	0.46*	0.64*	0.65*	0.54*
	(0.45; 0.48)	(0.62; 0.66)	(0.53; 0.56)	(0.52; 0.56)
Unknown	0.63*	0.81*	0.69*	0.71*
	(0.60; 0.66)	(0.77; 0.85)	(0.66; 0.72)	(0.68; 0.74)

Table notes. Raven: Raven progressive matrices test; Arith.: Arithmetic performance test; Language: language performance test. Significance: $^{*}p < 0.05$
